# *MBL2* gene polymorphisms in HHV-8 infection in people living with HIV/AIDS

**DOI:** 10.1186/s12977-018-0456-8

**Published:** 2018-11-27

**Authors:** Viviane Martha Santos de Morais, Elker Lene Santos de Lima, Georgea Gertrudes de Oliveira Mendes Cahú, Thaisa Regina Rocha Lopes, Juliana Prado Gonçales, Maria Tereza Cartaxo Muniz, Maria Rosângela Cunha Duarte Coêlho

**Affiliations:** 1 Virology Division, Laboratory of Immunopathology Keizo Asami (LIKA), Federal University of Pernambuco, Av. Prof. Moraes Rego, 1235, Cidade Universitária, Recife, PE 50670-901 Brazil; 2 Laboratory of Molecular Biology, Center of Pediatric Oncohematology, Oswaldo Cruz University Hospital, University of Pernambuco, Recife, PE Brazil; 3 Departament of Physiology and Pharmacology, Center of Biological Sciences, Federal University of Pernambuco, Recife, PE Brazil

**Keywords:** Human herpesvirus 8, Mannose binding lectin, HIV/HHV-8 coinfection, *MBL2* gene

## Abstract

**Background:**

Host genetic factors such as *MBL2* gene polymorphisms cause defects in the polymerization of MBL protein and result in a functional deficiency and/or in low serum levels that can influence susceptibility to various viral infections. The aim of this study was to estimate the frequency of alleles, genotypes and haplotypes related to -550, -221 and exon 1 polymorphisms of the *MBL2* gene and investigate their association with HHV-8 in people living with HIV/AIDS (PLWHA), as well as the impacts on CD4 cell count and HIV viral load in HIV/HHV-8 coinfected and HIV monoinfected patients.

**Results:**

A cross sectional study in PLWHA, with and without HHV-8 infection, exploring associations between different factors, was performed in the outpatient infectious and parasitic diseases clinic at a referral hospital. Genomic DNA extractions from leukocytes were performed using a commercial *Wizard*^*®*^ *Genomic DNA Purification* kit (Promega, Madison, WI). The promoter region (-550 and -221) was genotyped with the TaqMan system (Applied TaqMan Biosystems^®^ genotyping Assays), and the structural region (exon1) was genotyped with *Express Sybr Greener Supermix* kit (Invitrogen, USA). In total, 124 HIV/HHV-8 coinfected and 213 HIV monoinfected patients were analysed. Median TCD4 counts were significantly lower in HIV/HHV-8 coinfected patients, whereas the mean of the first and last viral load of HIV did not present significant difference. There was no difference in frequency between the LL, YY and AA genotypes between the HIV/HHV-8 coinfected or HIV monoinfected patients. However, in a multivariate analysis, coinfected patients with the intermediate expression haplotype of the *MBL2* gene had an odds ratio of 3.1-fold (CI = 1.2–7.6) of their last CD4 cell count being below 350 cells/mm^3^. Among the coinfected individuals, four developed KS and presented the intermediate expression *MBL* haplotype, with three being HYA/LXA and one being LYA/LYO.

**Conclusions:**

Host genetic factors, such as -550, -221 and exon 1 polymorphisms, can be related to the may modify coinfections and/or to the development clinical manifestations caused by HHV-8, especially in HIV/HHV-8 coinfected patients who present the intermediate expression haplotypes of MBL.

## Background

Human herpesvirus 8 (HHV-8) is associated with all forms of Kaposi’s sarcoma (KS), being the necessary aetiological agent but insufficient alone for the development of this disease [1, 2]. Therefore, other factors such as host genetic polymorphisms may influence the development of clinical manifestations caused by HHV8. The prevalence of HHV-8 varies according to geographic regions and sub-populations, but after the human immunodeficiency virus (HIV) epidemic, the incidence of KS increased drastically compared to HIV-negative individuals, being one of the commonest cancers in people living with HIV (PLWHA) [3–6]. These coinfected patients have a more aggressive clinical course and progression to death [3–6].

The host’s innate and adaptive immune responses have a key role in the control of HHV-8 infection and the early stages of KS development [7–9]. Mannose-binding lectin (MBL) is a protein of the innate immune system that binds with high affinity to mannose or other carbohydrates that may be present on the surface of the pathogens. MBL can mediate phagocytosis by macrophages, and when associated with serine protease (MASP), it activates the complement system by the lectin pathway in an antibody-independent mechanism [10, 11].

The protein expression of MBL is determined genetically, and the serum concentration is directly related to mutations in the structural region of the *MBL2* gene (exon 1) and in other polymorphic sites in the promoter region (-550 and -221). These three loci are closely related, and the allelic combinations of these regions result in different haplotypes that can cause defects in the polymerization of the molecule resulting in a functional deficiency and/or in low serum levels of MBL [12–16]. Thus, these polymorphisms have been associated with susceptibility or resistance to viral infections or the development of clinical manifestations and were studied in patients with hepatitis C virus, hepatitis B virus, human papillomavirus, Epstein-Barr virus, and dengue virus [17–27].

The progression of the disease caused by HIV infection is characterized by a decline in TCD4 cell count and an increase in viral load, leading to patient death in the absence of antiretroviral therapy [28]. Thus, some studies suggest that in HIV/HHV-8 coinfection, severe depletion or inactivation of T cells and HIV replication may be important factors in the clinical evolution of HHV-8 infection and the development of KS [4, 29–31].

However, host genetic factors such as *MBL2* gene polymorphisms can control HIV/HHV-8 coinfection and influence on HHV-8 infection in PLWHA and/or the clinical evolution of coinfected individuals. The aim of this study was to estimate the frequency of alleles, genotypes and haplotypes related to -550, -221 and exon 1 polymorphisms of the *MBL2* gene in HIV/HHV-8 coinfected and HIV monoinfected patients. In addition, we also explored associations of these gene variants with TCD4 cell count and HIV viral load in both groups.

## Methodology

### Study population

This is a cross section of PLWHA, with and without HHV-8 infection, in which patients’ demographic and laboratory variable data were originally described by Cahú et al. [32] in a research on the HIV/HHV-8 coinfection prevalence in 500 PLWHA, reporting 143 HIV/HHV-8 coinfected and 357 HIV monoinfected. Of this total, we randomly selected 124 HIV/HHV-8 coinfected and 213 HIV monoinfected patients, by the statistical program Epi Info version 7.1.5 (CDC, Atlanta, GA, USA), who were followed up by the doctors from the outpatient clinic Infectious and Parasitic Diseases in the Clinical Hospital of the Federal University of Pernambuco. This sample size was calculated using the same software, with the 99.9% confidence interval, with study power of 80%, prevalence of exposure to gene polymorphism *MBL2* of 17%. At the time of initial measurement all patients were on ART and there were no patients with KS, as evaluated in the medical records and through an interview with the patient. This research was approved by the ethics committee and the institutional review board of the Federal University of Pernambuco under case number 22428813.5.0000.5208.

### DNA extraction and genotyping

Genomic DNA was extracted from leukocytes isolated from whole blood in anticoagulant solution (EDTA) using a commercial kit *Wizard*^*®*^ *Genomic DNA Purification* (Promega, Madison, WI) following the manufacturer’s instructions. After extraction, all samples were measured to determine the concentration and the degree of purity of DNA using the Thermo Scientific NanoDrop 2000 spectrophotometer. All samples were diluted to the DNA concentration required for each reaction.

The promoter region (-550 and -221) was genotyped using the TaqMan system (Applied Biosystems^®^ Genotyping Assays TaqMan), and the structural region (exon 1) genotyping was performed using the Express *Sybr Greener Supermix* kit (Invitrogen, USA). The melting temperature (melting temperature assay, MTA) and the melting curve profiles were obtained using the decoupling device software. The detection of polymorphisms in the codons -550, -221 and the exon 1 *MBL2* gene was performed using the Rotor Gene 6000 (Corbett Research, Sydney, Australia). The three allelic variants of the *MBL2* gene in codon positions 52, 54, and 57 in exon-1 were designated ‘O’, and the wild-type allele was designated ‘A’, as previously described in several studies [21, 27, 33, 34], the sequences of the primers and probes used are described in Table 1.

**Table 1:** Sequence of primers and probes used to detect polymorphisms of the promoter and structural regions of the *MBL2* gene

*MBL2* gene region	Nucleotide sequence
Promoter region (-550)	
Primer forward	5′-CCAACGTAGTAAGAAATTTCCAGAGA-3′
Primer reverse	5′-CAACCCAGCCCAGAATTAACTG-3′
Sonda VIC	5′-VIC-CCTGTCTAAAACACC-MGB-3
Sonda FAM	5′-FAM-AGCCTGTGTAAAAC-MGB-3
Promoter region (-221)	
Primer forward	5′-GCACGGTCCCATTTGTTCTCA-3′
Primer reverse	5′-GCGTTGCTGCTGGAAGACTATAAA-3′
Sonda VIC	5′-VIC-CATGCTTTCGGTGGCAG-MGB-3′
Sonda FAM	5′-FAM-CATGCTTTCCGTGGCAG-MGB-3′
Structural region (exon-1)	
Primer forward	5′-AGGCATCAACGGCTTCCCA-3′
Primer reverse	5′-CAGAACAGCCCAACACGTACCT-3′

### *MBL2* gene haplotypes according to the grouping of the genotypes

The genotypes of the promoter region (-550 H/L and -221 Y/X) were grouped into the genotype of the structural region (exon 1 A/O) and categorized according to the haplotypes. We considered high expression haplotypes to be LYA/LYA, HYA/HYA and HYA/LYA. Intermediate expression haplotypes to be HYA/HXA, HYA/HYO, HYA/LXA, HYA/LYO, LXA/LXA, LYA/LXA and LYA/LYO. Low expression haplotypes to be HYO/HYO, LYO/LYO and HYO/LYO, according to some studies [27, 34, 35].

### Statistical analysis

For the statistical analysis, we used Epi Info version 7.1.5 (CDC, Atlanta, GA, USA) and Rstudio version 1.0.143 (Northern Ave, Boston, MA) and considered values of *p* < 0.05 as statistically significant. Additionally, the values of the odds ratios (ORs) and confidence intervals (95% CIs) were used for each respective value of *p*. Categorical variables were analysed Chi-square test (χ^2^) or Fisher’s exact test, while continuous variables were analysed using Mann–Whitney and Kruskal–Wallis tests. To verify if the population was in Hardy–Weinberg equilibrium and for the construction of the haplotypical combination, we used the programme Arlequin version 3.11 (Institute of Ecology and Evolution, University of Bern).

## Results

In total, 337 samples of PLWHA were analysed, of which 124 were HIV/HHV-8 coinfected, and 213 were HIV monoinfected patients. Table 2 shows the frequency of the sociodemographic variables distributed between coinfected and monoinfected patients.

**Table 2:** Frequency of sociodemographic variables in coinfected and monoinfected patients

Variables	HIV/HHV-8 n = 124	HIV n = 213	*p* value^a^	OR (95% CI)
Age	42.8 (± 11.9)^b^	42.7 (± 11)^b^	0.9^c^	–
Sex				
Male	87 (70.2%)	131 (62.5%)	–	Reference
Female	37 (29.8%)	82 (38.5%)	0.1	1.5 (0.9–2.4)
Ethnicity^d^				
Mulattos	56 (45.2%)	101 (47.4%)	–	Reference
White	36 (29.0%)	60 (28.2%)	0.8	0.9 (0.5–1.6)
Black	30 (24.2%)	48 (22.5%)	0.8	0.9 (0.5–1.5)
Amerindians	1 (0.8%)	3 (1.4%)	1.0	1.7 (0.2–16.4)
Asians	1 (0.8%)	1 (0.5%)	1.0	0.5 (0.03–9.0)

*OR* Odds ratio, *CI* confidence interval

^a^*p* values Chi-square test or Fisher’s exact test

^b^Values referring to mean age with the standard deviation indicated in parentheses

^c^*p* values ANOVA test

^d^Based on ethnic self-identification, according to the classification system of the Brazilian Institute of Geography and Statistics

Table 3 shows the median values of the clinical variables analysed in coinfected and monoinfected patients.

**Table 3:** Median values of the clinical variables analysed in coinfected and monoinfected patients

Median of variables	HIV/HHV-8 n = 124 (range)^b^	HIV n = 213 (range)^b^	*p* value^a^
First count of TCD4 cell (cell/mm^3^)	177.5 (4–1206)	275 (4–1565)	*0.01*
Last count of TCD4 cell (cell/mm^3^)	525 (20–1674)	573 (15–2171)	0.8
First viral load of HIV (copies/ml)	23.859 (79–4.500.0)	11.892 (49–1.642.2)	0.3
Last viral load of HIV (copies/ml)	< 40* (< 40–346.3)	< 40* (< 40–1.873.5)	0.7
Time of HIV infection (years)	6.8 (0–24.2)	5.7 (0–25.2)	0.3
Time from HIV diagnosis to the start of ART (years)	0.29 (0–20.2)	0.28 (0–20.0)	0.7
Time on ART (years)	4.7 (0–18.0)	4.1 (0–18.0)	0.2

*All were on ART. The table has no missing data

^a^*p* values Kruskal–Wallis test

^b^Minimum and maximum values are referenced in bracket

The frequencies of the alleles, genotypes and haplotypes related to -550, -221 and exon 1 polymorphisms of the *MBL2* gene are shown in Table 4. According to the Hardy–Weinberg test, the groups were balanced.

**Table 4:** Distribution of frequencies of alleles, genotypes and haplotypes of the *MBL2* gene between monoinfected and coinfected patients

Variables	HIV/HHV-8 n = 124 (%)	HIV n = 213 (%)	*p* value^a^	OR (95% CI)
Promoter (-550)				
Alleles				
L	173 (70)	297 (70)	–	Reference
H	75 (30)	129 (30)	1.0	1.0 (0.7–1.4)
Genotypes				
LL	62 (50)	102 (48)	–	Reference
HL	49 (40)	93 (44)	0.5	1.1 (0.7–1.8)
HH	13 (10)	18 (8)	0.7	0.8 (0.4–1.8)
Promoter (-221)				
Alleles				
Y	210 (85)	356 (84)	–	Reference
X	38 (15)	70 (16)	0.7	1.1 (0.7–1.7)
Genotypes				
YY	89 (72)	148 (70)	–	Reference
YX	32 (26)	60 (28)	0.6	1.1 (0.7–1.9)
XX	3 (2)	5 (2)	0.6	1.0 (0.2–4.3)
Exon-1 (52, 54, 57)				
Alleles				
A	196 (79)	344 (81)	–	Reference
O	52 (21)	82 (19)	0.6	0.9 (0.6–1.3)
Genotypes				
AA	81 (65)	143 (67)	–	Reference
AO	34 (28)	58 (27)	0.9	1.0 (0.6–1.6)
OO	9 (7)	12 (6)	0.5	0.7(0.3–1.9)
Haplotypes				
High expression	46 (37)	78 (36)	–	Reference
Intermediate expression	69 (56)	123 (58)	0.8	1.0 (0.7–1.7)
Low expression	9 (7)	12 (6)	0.6	0.8 (0.3–2.0)

*OR* Odds ratio, *CI* confidence interval

^a^*p* values Chi-square test or Fisher’s exact test

The frequency of haplotypes according to expression in coinfected and monoinfected patients is described in Table 5.

**Table 5:** Distribution of *MBL2* gene haplotype frequencies according to coinfected and monoinfected expression levels

Haplotypes	Expression	HIV/HHV-8 n = 124 (%)	HIV n = 213 (%)	*p* value^a^	OR (95% CI)
HYA/LYA	High	17 (13.7)	43 (20.2)	1	Reference
LYA/LYA	High	19 (15.3)	24 (11.3)	0.1	0.5 (0.2–1.1)
HYA/HYA	High	10 (8.1)	11 (5.2)	0.1	0.4 (0.1–1.2)
HYA/HXA	Intermediate	0 (0)	1 (0.5)	0.5	–
HYA/HYO	Intermediate	1 (0.8)	5 (2.3)	0.7	2.0 (0.2–18.2)
HYA/LXA	Intermediate	12 (9.7)	23 (10.8)	0.5	0.8 (0.3–1.9)
HYA/LYO	Intermediate	19 (15.3)	24 (11.3)	0.1	0.5 (0.2–1.14)
LYA/LXA	Intermediate	20 (16.1)	36 (16.9)	0.4	0.7 (0.3–1.6)
LYA/LYO	Intermediate	14 (11.3)	29 (13.6)	0.6	0.8 (0.3–1.9)
LXA/LXA	Intermediate	3 (2.4)	5 (2.3)	0.7	0.7 (0.1–3.1)
HYO/HYO	Low	2 (1.6)	1 (0.5)	0.2	0.2 (0.02–2.3)
HYO/LYO	Low	1 (0.8)	3 (1.4)	1.0	1.2 (0.1–12.2)
LYO/LYO	Low	6 (4.8)	8 (3.8)	0.3	0.5 (0.2–1.7)

*OR* Odds ratio, *CI* confidence interval

^a^*p* values Chi-square test or Fisher’s exact test

Table 6 shows the frequencies of viral load of HIV and TCD4 counts according to high, intermediate and low expression levels of MBL in coinfected and monoinfected patients.

**Table 6:** Univariate analysis of HIV viral load and TCD4 counts according to the expression of haplotypes in coinfected and monoinfected patients

Variables	HIV/HHV-8 n = 124	HIV n = 213	*p* value^a^	OR (95% CI)
High expression haplotype				
First HIV viral load > 150,000	16 (34.8)	14 (17.9)	–	Reference
First HIV viral load ≤ 150,000	30 (65.2)	64 (82.1)	*0.03*	*2.4 (1.1–5.6)*
Last HIV viral load detectable	14 (30.4)	33 (42.3)	–	Reference
Last HIV viral load undetectable	32 (69.6)	45 (57.7)	0.2	0.6 (0.3–1.3)
First TCD4 cell count ≤ 350	34 (73.9)	41 (52.6)	–	Reference
First TCD4 cellcount > 350	12 (26.1)	37 (47.4)	*0.02*	*2.6 (1.2–5.7)*
Last TCD4 cell count ≤ 350	3 (6.5)	8 (10.3)	–	Reference
Last TCD4 cell count > 350	43 (93.5)	70 (89.7)	0.5	0.6 (0.1–2.4)
Intermediate expression haplotype				
First HIV viral load > 150,000	15 (21.7)	23 (18.7)	–	Reference
First HIV viral load ≤ 150,000	54 (78.3)	100 (81.3)	0.6	1.2 (0.6–2.5)
Last HIV viral load detectable	34 (49.3)	52 (42.3)	–	Reference
Last HIV viral load undetectable	35 (50.7)	71 (57.7)	0.3	1.3 (0.7–2.4)
First TCD4 cell count ≤ 350	52 (75.4)	76 (61.8)	–	Reference
First TCD4 cell count > 350	17 (24.6)	47 (38.2)	0.06	1.9 (1.0–3.6)
Last TCD4 cell count ≤ 350	15 (21.7)	9 (7.3)	–	Reference
Last TCD4 cell count > 350	54 (78.3)	114 (92.7)	*0.00*	*3.5 (1.4–8.5)*
Low expression haplotype				
First HIV viral load > 150,000	2 (22.2)	3 (25)	–	Reference
First HIV viral load ≤ 150,000	7 (77.8)	9 (75)	1.0	0.9 (0.1–6.6)
Last HIV viral load detectable	5 (55.6)	8 (66.7)	–	Reference
Last HIV viral load undetectable	4 (44.4)	4 (33.3)	0.7	0.6 (0.1–3.7)
First TCD4 cell count ≤ 350	6 (66.7)	10 (83.3)	–	Reference
First TCD4 cell count > 350	3 (33.3)	2 (16.7)	0.6	0.4 (0.1–3.1)
Last TCD4 cell count ≤ 350	3 (33.3)	3 (25)	–	Reference
Last TCD4 cell count > 350	6 (66.7)	9 (75)	1.0	1.5 (0.2–10.1)

*OR* Odds ratio, *CI* confidence interval

^a^*p* values Chi-square test or Fisher’s exact test

The variable last TCD4 count in coinfected patients with intermediate expression haplotype remained associated following the final model in the multivariate analysis, as is shown in Table 7 with the adjusted odds ratio (OR) and confidence intervals (95% CI).

**Table 7:** Multivariate analysis of HIV viral load and TCD4 cell count according to the expression of haplotypes in coinfected and monoinfected patients

Variables	HIV/HHV-8 n = 124	HIV n = 213	*p* value^a^	OR (95% CI)
High expression haplotype				
First HIV viral load > 150,000	16 (34.8)	14 (17.9)	–	Reference
First HIV viral load ≤ 150,000	30 (65.2)	64 (82.1)	0.1	2.1 (0.9–5.1)
Last HIV viral load detectable	6 (13)	17 (21.8)	–	Reference
Last HIV viral load undetectable	40 (87)	61 (78.2)	0.2	0.4 (0.1–1.4)
First TCD4 cell count ≤ 350	34 (73.9)	41 (52.6)	–	Reference
First TCD4 cell count > 350	12 (26.1)	37 (47.4)	0.1	2.0 (0.9–4.7)
Intermediate expression haplotype				
First TCD4 cell count ≤ 350	52 (75.4)	76 (61.8)	–	Reference
First TCD4 cell count > 350	17 (24.6)	47 (38.2)	0.2	1.6 (0.8–3.1)
Last TCD4 cell count ≤ 350	15 (21.7)	9 (7.3)	–	Reference
Last TCD4 cell count > 350	54 (78.3)	114 (92.7)	*0.02*	3.1 (1.2–7.6)

*OR* Odds ratio, *CI* confidence interval

^a^*p* values Chi-square test

Among the coinfected, four developed KS during the clinical course of HIV/HHV-8 coinfection and all were characterized as intermediate expression haplotypes, three were HYA/LXA and one LYA/LYO.

## Discussion

The pathogenesis of HIV/HHV-8 coinfection is complex and can be influenced by viral factors; for example, HIV induces the HHV-8 lytic cycle through the activation of the RTA protein and the TAT protein, and HHV-8 interferes with HIV replication by regulating the LTR by LANA antigen [36–40]. However, host genetic factors such as *MBL2* gene polymorphisms may also influence factors as diverse as susceptibility to HHV-8 infection, as well as impact on important factors such as HIV viral load and TCD4 counts.

The results of our research showed that the median of the first TCD4 cell count was lower in coinfected patients than monoinfected patients with a statistically significant difference. However, another study has shown that HHV-8 has little influence on HIV progression in initially asymptomatic individuals, with little repercussion in TCD4 cell count [41]. Nonetheless, considering that our study was a cross section of PLWHA, with or without HHV-8 infection, it was not possible to identify the moment of HHV-8 seroconversion, because ours was not an observational cohort study. Highlighting the importance of more specific studies with HIV/HHV-8 coinfected patients.

The median of the last TCD4 count was not statistically significant between the coinfected and monoinfected patients, corroborating with some previous studies [38, 39]. Additionally, another study did not find an association between coinfection in PLWHA with KS and the last TCD4 count [42]. The absence of an association in PLWHA with and without KS may suggest that factors other than TCD4 influence the pathogenesis of HIV/HHV-8 coinfection. Similarly, the median values of the first and last HIV viral load did not present statistically significant differences between the groups. However, the coinfected patients had a median value of the first viral load two times higher, however did not present statistically significant differences.

Some studies have shown that the order and timing of HIV and HHV-8 infection may have prognostic implications and that the incidence of KS is higher in people with HHV-8 seroconversion following HIV infection [3, 38, 43–45]. In the present study, the time of HIV infection as well as the time from diagnosis to the initiation of antiretroviral therapy (ART) was lower in coinfected patients, although not statistically significant. This corroborates a study that evaluated the time of seroconversion for HHV-8 and did not find an association between HHV-8 infection and HIV disease progression the time of diagnosis and the need to use ART [38], suggesting that HHV-8 does not accelerate the need to use ART.

The importance of the host genetic factors such as *MBL2* gene polymorphisms and its association with infections has been studied for several viruses [17–21, 25–27]. In this study, the frequency of the alleles, genotypes and haplotypes of -550, -221 and exon 1 polymorphisms of the *MBL2* gene had no association with HHV-8 infection in PLWHA. However, it is important to highlight there is very little research in this area to compare our results.

One study evaluated the -550, -221 and exon 1 polymorphisms in PLWHA and found an association between the LX/LX genotype and low expression haplotypes with HIV infection when compared with blood donors. However, the authors reported that the study population was composed of PLWHA of European ethnicity and suggested that individuals of this origin may be more susceptible to HIV infection [46]. Nonetheless, a study of Zimbabweans in South Africa found no association of these polymorphisms with HIV infection when compared to uninfected individuals [47]. Most of the individuals evaluated in the present study declared themselves mulattos, which may have contributed to the non-association with HHV-8 infection in PLWHA. The absence of an association may be related to the ethnic groups of Northeast Brazil, which have a combined mixture of the genomes of Africans, Europeans and Native Americans [48–50].

Simultaneous analysis of the -550, -221 and exon 1 polymorphisms can provide more complete information on the role of polymorphisms in infections since the concentrations of MBL may vary depending on the combinations of the structural and promoter polymorphisms [12–14]. According to Vallinoto et al. [51], these polymorphisms may be genetic markers associated with a better response in HIV-infected individuals using older antiretroviral therapy regimens. When exon 1 polymorphism was studied in isolation, the presence of the A allele was associated with the reduction of HIV viral load and the improvement of the TCD4 count in PLWHA [51]. The high expression haplotype HY, referring to codons -550 and -221, was associated with a decrease in viral load and an increase in TCD4 counts during the clinical course of monoinfected HIV patients [52].

In contrast, the -550, -221 and exon 1 polymorphisms were studied simultaneously in PLWHA and showed no impact on viral load or TCD4 count [53], and they were also not associated with viral load, TCD4 count, disease progression, or survival of a population of PLWHA from Africa who was not under antiretroviral therapy [28]. However, the present study simultaneously evaluated the three main polymorphisms of the *MBL2* gene and the high, intermediate and low expression haplotypes and found an association between the HIV/HHV-8 coinfected patients who had the intermediate expression haplotype with TCD4 count when compared with patients monoinfected by HIV.

Considering the ability of MBL to bind to the HIV-1 gp120 glycoprotein and mediating phagocytosis or activating the complement system [54–56], our results demonstrate that coinfected patients who present the intermediate expression haplotype of *MBL2* gene may have the last TCD4 count < 350 cells/mm^3^. It is important to note that among the coinfected patients, the four who developed KS also had intermediate expression MBL haplotypes, three with HYA/LXA and one with LYA/LYO, suggesting that these haplotypes may interfere with the clinic development of SK in HIV/HHV-8 coinfected patients. It should also be emphasized that none of the coinfected patients who presented the high expression haplotypes of *MBL2* gene developed KS during clinical follow-up.

The role of *MBL2* gene polymorphisms is still a subject that needs to be further studied both in HHV-8 infected and HIV/HHV-8 coinfected patients, mainly in other populations of regions endemic to HHV-8, due to the importance of MBL protein in the innate immune system. Host genetic factors such as -550, -221 and exon 1 polymorphisms cause defects in the polymerization of MBL protein, resulting in a functional deficiency and/or in low serum levels of this protein that can influence the individual susceptibility to various viral infections. Therefore, these polymorphisms can be related to the coinfection and/or to the development clinical manifestations caused by HHV-8, as the KS, especially in HIV/HHV-8 coinfected patients who present the intermediate expression haplotypes of MBL.
